# A randomised, double-blind, placebo-controlled trial of a multi-strain probiotic in patients with asymptomatic ulcerative colitis and Crohn’s disease

**DOI:** 10.1007/s10787-019-00595-4

**Published:** 2019-05-03

**Authors:** Ingvar Bjarnason, Guy Sission, Bu’Hussaine Hayee

**Affiliations:** 1 Departments of Gastroenterology At King’S College Hospital, Denmark Hill, London, SE59RS UK; 2 Darent Valley Hospital, Darent Wood Road, Dartford, Kent UK

**Keywords:** Complementary, Alternative therapy, IBD, Probiotic

## Abstract

**Background:**

There is considerable interest in the possible importance of the gut microflora in the pathophysiology of the inflammatory bowel diseases (IBD) ulcerative colitis (UC) and Crohn’s disease (CD). Probiotics offer a potential adjuvant treatment in these patients by modifying the intestinal milieu, but reports of their efficacy are conflicting.

**Aims:**

To assess the efficacy of a multi-strain probiotic (Symprove™, Symprove Ltd, Farnham, United Kingdom) in quality of life issues and intestinal inflammation in patients with asymptomatic UC and CD.

**Methods:**

A single-centre, randomised, double-blind, placebo-controlled trial of adult patients with asymptomatic IBD. Patients received 4 weeks of treatment with the probiotic or placebo (1 ml/kg/day). The primary efficacy measure was the difference in change in the IBD Quality of Life Questionnaire results (QOL) between probiotic vs. placebo at week 4. Secondary outcome measures included analyses of the change in laboratory findings, including faecal calprotectin (FCAL).

**Results:**

Over 500 patients were recruited to the study and 81 and 61 patients with UC and CD, respectively were randomised and completed the study. There were no significant differences in IBD-QOL scores between placebo and the probiotic groups. Similarly, there were no significant changes observed in the laboratory data. However, the differences in FCAL between patients with UC before and after probiotics versus placebo approached statistical significance with a *p* value of 0.076. Post-hoc analyses showed that the FCAL levels were significantly (*p* < 0.015) reduced in the UC patients receiving the probiotic as opposed to placebo. No significant changes were seen in CD. No serious adverse events were observed.

**Conclusion:**

This multi-strain probiotic is associated with decreased intestinal inflammation in patients with UC, but not in CD and is well tolerated. Further research is required to see if the probiotic reduces the incidence of clinical relapses in asymptomatic IBD patients.

## Introduction

New medical treatments are emerging for the treatment of the inflammatory bowel disease (IBD) ulcerative colitis (UC) and Crohn’s disease (CD) and the positioning of established medication is continuously being refined. Most of the new treatments are for severe disease, but patients continue to experiment with non-drug related remedies in the hope of improving wellbeing. Accordingly many people with IBD seek means of altering lifestyle issues including complementary alternative treatment (Hilsden et al. 1999, 2003; Quattropani et al. 2003) and they frequently do so without consulting their doctors (Bensoussan et al. 2006). The most common alternative treatments are homeopathy, acupuncture, traditional Chinese medicine and increasingly the use of probiotics (Joos et al. 2006).

The interest in probiotics has been fueled by new technologies that demonstrate that the intestinal microbiome is significantly altered in a number of intestinal and extra-intestinal diseases, suggesting that bacterial dysbiosis may have an etiological or pathogenic role in some of these disorders (Ni et al. 2017). This is of particular relevance in UC and CD as it offers the possibility that probiotics might be useful as an adjuvant treatment.

Antimicrobials have an established albeit limited place in the treatment of IBD, but the use of other regimes intended to interact with the intestinal flora, such as colonic lavage, dietary modifications (Obih et al. 2016), faecal microbiota transplants (Cohen and Maharshak 2017) and probiotics (Weizman et al. 2012) are much more controversial (Cheifetz et al. 2017). The main problem for acceptance of some of these studies is that the trials have lacked controls, etc. Indeed there are no regulatory requirements for randomized controlled trials when it comes to food supplements (probiotics), dietary management and similar interventions (Gallinger et al. 2014; Cheifetz et al. 2017).

Prebiotics have nevertheless been assessed in IBD in order to reduce symptoms in active disease and to prolong clinical remission. The perception from Cochrane and other reviews are that some probiotics [*E. coli* (Nissle 1917)], VSL-3, *Bifidobacterium longum* (along with a pre-biotic fructo-oigosaccharide/inulin mix), *Bifidobacteria* fermented milk) reduce clinical disease activity modestly, using a variety of comparators and outcome measures (Mallon et al. 2007). Some [VSL-3 (2 studies), *Lactobacillus* GG, *E. coli* (Nissle 1917) (5 studies), *Bifidobacterium*, *Bifidobacterium longum*, *Bifidobacterium breve* and *bifidum* with *Lactobacillus*, *Bifidobacterium breve* and *longum* (Yakoult with *Lactobacillus*)] may prolong remission (Zigra et al. 2007; Sang et al. 2010) in patients with mild to moderately severe UC (when added to standard therapy), compared with a variety of comparators and outcome measures. However, probiotics [Lactobacillus GG (4 studies) Lactobacillus johnsonii (2 studies), VSL-3 and *E. coli* (Nissle 1917)] do not prolong remission significantly in CD (Rolfe et al. 2006; Rahimi et al. 2008; Doherty et al. 2009; Shen et al. 2009), again using various outcome measures.

Probiotics differ in their formulation including bacteriological composition so that the effect of a particular brand cannot be extrapolated over to another one. Symprove is a liquid based probiotic containing live bacteria that survive a simulated gastrointestinal environment (Fredua-Agyeman and Gaisford 2015). Previous trials using Symprove show a significant improvement in the overall irritable bowel syndrome (IBS) symptom severity score (mostly abdominal pain and bowel habits) in patients with IBS (Sisson et al. 2014) and some symptoms associated with diverticular disease (constipation, diarrhoea, mucorrhoea and back pain) (Kvasnovsky et al. 2017). Symprove had a tendency to reduce intestinal inflammation in IBS and may prevent inflammation in diverticular disease.

Our purpose was to assess the short-term effects of Symprove on quality of life and intestinal inflammation in a cohort of asymptomatic patients with IBD as compared with placebo.

## Material and methods

This was a single-centre, randomized, double-blind, placebo-controlled trial designed to assess the short-term efficacy of the probiotic Symprove to improve quality of life and alter intestinal inflammation in patients with quiescent or mild symptoms of UC and CD (IORAS: 183662). The trial took place at King’s College Hospital, London, between November 2010 and October 2014.

The study was conducted in accordance with the guidelines for Good Clinical Practice (CPMP ICH 135 95), the principles of the Declaration of Helsinki and with all relevant local and national guidelines including the archiving of records. All patients were provided with written and verbal information about the study and subsequently gave informed written consent before study entry.

The Medicines and Healthcare products Regulatory Agency was consulted prior to starting this study and concluded that Symprove is classified as a food supplement rather than an investigational medicinal product.

The National Research Ethics Service approved the study protocol. Further review was undertaken locally by the Research and Development Committee of King's College Hospital who acted as Sponsors for the study.

Patients with UC and CD were recruited from King’s College Hospital Gastroenterology outpatients. The main inclusion criteria were patients attending routine clinical review with established UC and CD, age 18–70 years, diagnosed at least 6 months prior to the trial. Patients were required to have stabile inactive clinical disease, as defined by < 5 points on Harvey Bradshaw score (Harvey and Bradshaw 1980) (which corresponded to a score of ≤ 4 on the Truelove-Witts criteria (Truelove and Witts 1955) for ulcerative colitis, without a change in medication for 4 months. Patients on no treatment, maintenance treatment with a 5-aminosalicylic preparation or low dose Azathioprine (1 mg/kg) were eligible for inclusion in the trial, but those on steroids (prednisolone > 4 mg/day) and biologics were excluded from the study.

Exclusion criteria included patients having undergone intestinal resection, patients with serious co-morbidity including neurological, rheumatological, respiratory, nephrological, cardiovascular, psychiatric disease, patients with alcohol or drug addiction or dependency problems (within the last 5 years) and pregnant or lactating women. Patients with previous intolerances or adverse reactions to probiotics or the use of these products within the preceding 3 months were excluded.

### Study protocol

Study participants were randomized using a two-stage computerized randomization protocol provided by the Department of Pharmacy at King’s College Hospital. One group received the probiotic and the other a matching placebo. The duration of the study was 4 weeks.

All patients underwent a clinical examination at the initial clinic visit with documentation of demographic details. Patients completed a brief general health questionnaire and Quality of Life Questionnaire (CLQ) and underwent full blood counts, renal and liver function, C-reactive protein (CRP), erythrocyte sedimentation rate (ESR) and faecal calprotectin (FCAL).

Blinding of allocation to treatment was maintained until the completed study data-base was locked and passed over to an independent study statistician (Dr. Jackie Turner, Premier Research, Mulberry Business Park, Fishponds Road, UK).

Compliance was assessed at the end of the study when patients were asked to whether they had missed “no dose”, less than one dose a week, one to three doses a week.

### Study intervention

Symprove (Symprove Ltd, Farnham, Surrey UK) is a dietary food supplement probiotic, which contains 4 strains of naturally-occurring bacteria: *Lactobacillus rhamnosus* NCIMB 30174, *Lactobacillus plantarum* NCIMB 30173*, Lactobacillus acidophilus* NCIMB 30175 and *Enterococcus faecium* NCIMB 30176 in a water-based suspension of barley extract with each 50 ml/dose containing about 10 billion live bacteria. The placebo was an identical liquid in appearance and taste, containing water and flavouring and was provided in identical packaging supplied by the manufacturers identified by a trial batch and code number only.

Patients were asked to keep the study medication refrigerated between 2 and 7 °C and to self-administer 1 mL/kg each morning on a fasting stomach. Foods and fluids were allowed 20 min later. Missed does could be taken later during the day provided that no food had been consumed during the preceding 3 h.

### Clinical outcomes

The primary efficacy measure was the change of overall IBD QOL which involves 32 items (Guyatt et al. 1989) relating to 4 aspects of the patients lives; namely symptoms related to IBD; systemic symptoms; emotional and social function.

Secondary measures were the differences in clinical disease activity scores between active and placebo treatment and changes in laboratory measures including FCAL (EK-CAL, Buhlmann, Switzerland).

### Statistics

All statistical analyses was carried out by using SAS Software version 9.3 or later. Given the exploratory nature of the study and the fact that Symprove had not been studied in patients with IBD the sample size (40 patients in each group) was calculated on the assumption that 60% of patients would respond with a 30% response in the placebo group which gives an 80% power at a 5% significance level.

The efficacy measures were analysed on an intention-to-treat basis which included all patients randomized to any treatment. A *p* value of at or below 0.05 was considered as a statistically significant result.

For dichotomous and categorical variables, absolute and relative frequencies (counts and percent) are presented. In general, the denominator for the percentage calculation is be based upon the total number of patients in the respective study population, unless otherwise specified.

For continuous variables, comprehensive data summaries are presented with sample characteristics [number of non-missing observations (N), arithmetic mean, standard deviation (SD), minimum, lower quartile, median, upper quartile and maximum] by treatment. Where data were collected over time, both the observed data and the change from baseline are summarized at each visit.

The IBD QOL score was derived using the weightings given as previously described (Cheung et al. 2000), with the difference between treatment groups tested using an analysis of covariance on the value at week 4, with the baseline value as the covariate. Further exploratory statistical analyses were done using the paired *T* test and Wilcoxon’s signed rank test.

## Results

Between November 2010 and October 2014 over 300 patients with UC and 250 patients with CD were considered for participation in these studies. The trial was terminated in November 2014 when no patient with CD had been recruited for 5 months.

No significant side effects were reported, and the probiotic was well tolerated by everyone.

Table 1 shows the Demographic details of the whole study group.

**Table 1:** Demographic details

	Ulcerative colitis		Crohn’s disease	
	Probiotic (*N* = 40)	Placebo (*N* = 41)	Probiotic (*N* = 33)	Placebo (*N* = 29)
Mean age ( ± SD) years	47.3 ± 14.4	43.4 ± 12.1	41.2 ± 13.0	39.0 ± 13.0
M/F ratio				
Treatment				
5 ASA	31	33	15	12
Azathioprine	2	2	4	2
Prednisolone	1	0	1	0
None	7	6	13	15
Disease location UC				
Proctosigmoid	19	21		
Left sided	9	10		
Pancolonic	12	9		
Disease location Crohn’s				
Small bowel			14	14
Colon			11	7
Small and large bowel			9	7

### Quality of life assessments

Table 2 shows the IBD QOL scores in patients with UC colitis and CD. The IBD QOL scores demonstrated uniformly good quality of life scores at entry to the study and remained so over the ensuing 4 weeks. Table 2 shows that the changes between patients, with UC or CD, on probiotics and placebo were not statistically significant for any of the parameters assessed.

**Table 2:** Inflammatory Bowel Disease Quality of Life

IBD quality of life	Mean baseline^a^	Mean at week 4^a^	Change: baseline to week 4^a^	Difference probiotic v placebo	*P* value
Ulcerative colitis					
Emotional symptoms					
Probiotic	13.4 ± 2.7	13.4 ± 2.6	− 0.1 ± 2.8		
Placebo	13.0 ± 3.0	13.3 ± 3.2	0.2 ± 2.6	− 0.4 (− 1.6, 0.5)	0.71
Bowel function-1					
Probiotic	13.9 ± 4.3	13.3 ± 5.0	− 1.1 ± 3.3		
Placebo	11.7 ± 3.8	11.8 ± 3.6	− 0.2 ± 3.1	− 1.0 (− 2.5, 0.6)	0.60
Social function					
Probiotic	10.9 ± 4.7	10.6 ± 4.6	− 0.3 ± 3.8		
Placebo	9.1 ± 3.0	8.8 ± 2.4	− 0.3 ± 2.8	0.0 (− 1.4, 1.4)	0.34
Bowel function-2					
Probiotic	8.6 ± 2.5	7.9 ± 2.8	− 0.8 ± 2.8		
Placebo	8.0 ± 2.7	8.8 ± 2.4	− 0.1 ± 2.2	− 0.6 (− 1.8, 0.5)	0.56
Systemic function					
Probiotic	10.9 ± 12.3	7.9 ± 2.8	− 0.6 ± 2.4		
Placebo	9.1 ± 3.0	8.0 ± 2.5	− 0.1 ± 2.0	− 0.5 (− 1.4, 0.4)	0.90
Crohn’s disease					
Emotional symptoms					
Probiotic	14.8 ± 4.5	14.0 ± 4.7	− 1.0 ± 3.0		
Placebo	15.3 ± 2.9	14.8 ± 3.9	− 0.3 ± 2.7	− 0.7 (− 2.4, 0.9)	0.34
Bowel function-1					
Probiotic	12.5 ± 4.1	12.5 ± 4.5	− 0.2 ± 3.0		
Placebo	12.4 ± 3.9	12.0 ± 4.6	− 0.4 ± 2.7	0.2 (− 1.4, 1.9)	0.78
Social function					
Probiotic	11.1 ± 4.8	10.5 ± 4.4	− 0.9 ± 2.7		
Placebo	10.8 ± 3.3	10.9 ± 4.0	0.0 ± 2.9	− 1.0 (− 2.4, 0.5)	0.22
Bowel function-2					
Probiotic	9.5 ± 2.8	8.5 ± 3.3	− 1.2 ± 2.3		
Placebo	9.1 ± 2.8	8.6 ± 2.6	− 0.5 ± 2.7	− 0.7 (− 2.1, 0.6)	0.36
Systemic function					
Probiotic	9.7 ± 2.5	9.7 ± 2.3	− 0.1 ± 1.3		
Placebo	9.5 ± 1.8	9.8 ± 2.0	0.2 ± 1.4	− 0.3 (− 1.0, 0.4)	0.49

Mean and (95% Confidence Interval)

^a^Mean SD

### Clinical disease activity

Table 3 shows the clinical disease activity scores in the patients with UC and CD, which were uniformly low at entry to the trial. The scores in the groups of patients did not differ significantly between the probiotic and placebo arms following treatment.

**Table 3:** Clinical disease activity before and after treatment

Harvey Bradshaw	Mean baseline ± SD	Mean ± SD at week 4	Mean change ( ± SD) baseline to week 4	Mean difference probiotic v placebo (95% CI)	*P* value
Ulcerative colitis					
Well being					
Probiotic	1.1 ± 0.9	0.8 ± 0.7	− 0.1 ± 1.0		
Placebo	0.9 ± 0.6	0.6 ± 0.7	− 0.2 ± 0.8	0.0 (− 0.3, 0.4)	0.31
Abdominal pain					
Probiotic	0.8 ± 0.8	0.5 ± 0.6	− 0.3 ± 0.8		
Placebo	0.8 ± 0.8	0.4 ± 0.5	− 0.3 ± 0.6	0.0 (− 0.2, 0.3)	0.49
Abdominal mass					
Probiotic	0.0 ± 0.0	0.0 ± 0.0	0.0 ± 0.0		
Placebo	0.0 ± 0.2	0.0 ± 0.0	0.0 ± 0.0	0.0 (0.0, 0.0)	1.0
Number of liquid stools					
Probiotic	2.6 ± 2.0	2.2 ± 2.3	− 0.1 ± 1.3		
Placebo	2.2 ± 2.2	1.8 ± 2.1	− 0.2 ± 0.9	0.2 (− 0.4, 0.8)	0.47
Complications					
Probiotic	0.1 ± 0.3	0.0 ± 0.0	− 0.0 ± 0.2		
Placebo	0.0 ± 0.2	0.0 ± 0.2	0.0 ± 0.0	− 0.1 (− 0.1, 0.0)	0.17
Total score					
Probiotic	4.4 ± 2.7	3.4 ± 3.1	− 0.6 ± 2.5		
Placebo	3.9 ± 2.7	2.8 ± 2.4	− 0.8 ± 1.5	0.3 (− 0.7, 1.3)	0.39
Crohn’s disease					
Well being					
Probiotic	1.0 ± 0.8	1.0 ± 0.9	0.0 ± 0.7		
Placebo	1.0 ± 0.8	1.0 ± 0.8	0.0 ± 0.6	0.0 (− 0.4, 0.4)	0.96
Abdominal pain					
Probiotic	1.0 ± 0.9	0.9 ± 0.9	− 0.1 ± 0.7		
Placebo	0.6 ± 0.7	0.6 ± 0.6	− 0.1 ± 0.5	− 0.1 (− 0.4, 0.2)	0.79
Abdominal mass					
Probiotic	0.0 ± 0.0	0.0 ± 0.0	0.0 ± 0.0		
Placebo	0.0 ± 0.0	0.0 ± 0.0	0.0 ± 0.0	0.0 (0.0, 0.0)	1.0
Number of liquid stools					
Probiotic	2.3 ± 2.0	2.1 ± 2.0	− 0.3 ± 1.4		
Placebo	2.3 ± 2.1	1.8 ± 0.8	− 0.5 ± 1.5	0.2 (− 0.5, 1.0)	0.53
Complications					
Probiotic	0.0 ± 0.0	0.0 ± 0.0	0.0 ± 0.0		
Placebo	0.1 ± 0.2	0.0 ± 0.2	0.0 ± 0.0	0.0 (0.0, 0.0)	1.0
Total score					
Probiotic	4.3 ± 3.0	3.5 ± 3.2	− 0.4 ± 2.2		
Placebo	4.0 ± 2.4	3.4 ± 2.5	− 0.6 ± 1.9	0.2 (− 1.0, 1.4)	0.66

Four patients experienced a clinical relapse during the study. All were on placebo and had calprotectin levels exceeding 250 µg/g.

### Laboratory data

Table 4 shows the haemoglobin, haematocrit, white blood cell count, ESR, CRP and FCAL results before and after treatment in patients with UC and CD. There were no significant changes in the blood-serological markers during treatment in the patients with UC or CD.

**Table 4:** Laboratory findings before and after treatment

	Mean baseline ± SD	Mean ± SD at week 4	Mean change ( ± SD) baseline to week 4	Mean difference probiotic v placebo (95% CI)	*P* value
Ulcerative colitis					
Haemoglobin (*n* < 12.0 g/dL)					
Probiotic	13.6 ± 2.1	13.6 ± 2.0	− 0.0 ± 0.8		
Placebo	13.7 ± 2.8	13.9 ± 2.5	0.1 ± 2.1	− 0.17 (− 0.88, 0.54)	0.59
Haemotocrit (*n* = 35–50%)					
Probiotic	42.4 ± 5.5	42.9 ± 5.1	0.3 ± 2.5		
Placebo	43.4 ± 5.2	43.2 ± 6.0	0.4 ± 2.9	1.06 (− 0.23, 2.36)	0.15
White blood cells (*n* = 4.5–11.0 × 10^9^/L					
Probiotic	7.3 ± 2.4	7.6 ± 2.6	0.1 ± 1.6		
Placebo	7.2 ± 2.1	7.3 ± 2.9	0.1 ± 2.5	− 0.08 (− 1.12, 0.97)	1.00
ESR (*n* = 0–22 mm/h)					
Probiotic	16.3 ± 14.1	17.5 ± 16.2	1.2 ± 8.2		
Placebo	13.4 ± 15.3	10.4 ± 12.1	0.1 ± 10.9	1.04 (− 4.31, 6.39)	0.52
CRP (*n* < 5 mg/L)					
Probiotic	8.5 ± 7.2	15.0 ± 24.4	7.2 ± 15.7		
Placebo	5.9 ± 3.6	17.8 ± 39.4	12.7 ± 45.8	− 5.50 (− 35.7, 24.7)	0.71
Calprotectin (*n* = < 50 µg/g)					
Probiotic	725 ± 726	411 ± 460	− 314 ± 719		
Placebo	436 ± 451	626 ± 1057	189 ± 981	− 503 (− 861, − 145)	0.078
Crohn’s disease					
Haemoglobin (*n* < 12.0 g/dL)					
Probiotic	13.5 ± 2.1	13.3 ± 1.4	− 0.2 ± 0.6		
Placebo	13.9 ± 1.6	14.0 ± 1.4	0.1 ± 0.9	− 0.26 (− 0.74. 0.22)	0.11
Haemotocrit (*n* = 35–50%)					
Probiotic	42.6 ± 4.5	42.1 ± 4.0	− 0.1 ± 2.4		
Placebo	42.9 ± 4.9	43.5 ± 3.9	0.4 ± 2.9	− 0.47 (− 2.1, 1.17)	0.26
White blood cells (*n* = 4.5 to 11.0 × 10^9^/L					
Probiotic	7.2 ± 3.1	7.1 ± 3.0	− 0.7 ± 1.5		
Placebo	8.2 ± 3.1	8.1 ± 2.2	0.4 ± 1.9	− 1.06 (− 2.13, 0.02)	0.04
ESR (*n* = 0-22 mm/h)					
Probiotic	12.3 ± 11.1	16.7 ± 11.1	− 2.7 ± 4.9		
Placebo	22.3 ± 14.0	13.3 ± 12.1	− 2.1 ± 2.9	− 0.54 (− 4.06, 2.98)	1.00
CRP (n < 5mg/L)					
Probiotic	11.8 ± 9.9	11.4 ± 9.4	− 0.2 ± 9.5		
Placebo	16.7 ± 16.9	9.3 ± 6.8	− 2.6 ± 7.1	2.35 (− 8.99, 13.69)	0.42
Calprotectin (*n* = < 50 µg/g)					
Probiotic	686 ± 982	525 ± 610	− 206 ± 1046		
Placebo	301 ± 279	549 ± 1011	289 ± 1012	− 496 (− 981, 10.4)	0.69

The differences in FCAL between patients with UC before and after probiotics did not demonstrate statistical significance with a *p* value of 0.076. A post hoc analyses was carried out using the paired *T* test and Wilcoxon’s signed rank tests to assess the changes within the group. This showed an imbalance in the calprotectin levels at baseline (higher in the probiotic group) in the patients with UC. Nevertheless, calprotectin levels were significantly decreased in the UC patients following 4 weeks in the probiotic group (*p* = 0.011 and 0.001, *t* test and Wilcoxon’s, respectively), whilst the levels increased non-significantly in the placebo group (*p* = 0.236 and 0.81, *t* test and Wilcoxon’s, respectively).

**Fig. 1:**
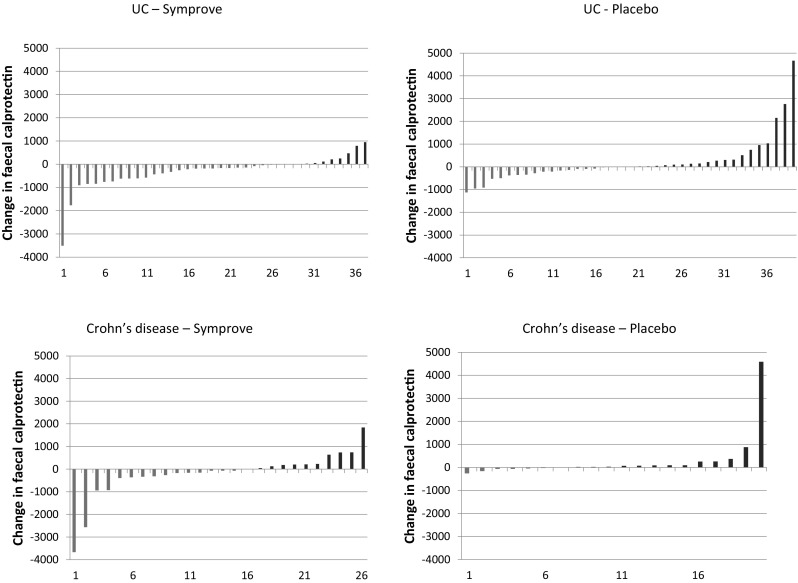
Individual change in FCAL levels in the study groups. The green bars indicate a reduction in FCAL levels whilst the red bars indicate an increase (color figure online)

The same analyses in the CD groups showed no statistically significant changes (*p* values ranged from 0.14–0.46). Figure 1 shows the individual changes in FCAL levels in patients with UC and CD during the trial.

## Discussion

This short-term, double-blind, randomized placebo-controlled clinical trial included patients with UC and CD that were in clinical remission and on minimal treatment. There were no significant changes in any of the measured outcome parameters, but there was a clear signal that the probiotic might be anti-inflammatory in patients with quiescent UC. This was supported by post-hoc analyses of the sequential changes of FCAL.

The medical management of IBD can be separated into two phases: induction and maintenance treatment (with a view to prolong the time to the next clinical relapse and reduce its severity). Maintenance treatment is advocated for most patients and this involves the administration of sulphasalazine or one of the 5-ASA preparations and at times azathioprine. The study group in the present study were predominantly clinically asymptomatic patients on 5 amino salicylic acid preparations and minimal other therapies, which might account for the uniformly good IBD QOL scores and the relatively normal laboratory parameters, apart from the FCAL levels. These did not change significantly during the treatments.

It is of note that, in patients otherwise in clinical remission, FCAL levels were increased in both UC and CD on entry to the trial, indicative of ongoing intestinal inflammation. This is a recognized finding in patients with IBD, but more importantly FCAL in excess of fivefold for the upper normal limit (e.g. 250 µg/g) confers a significant risk of clinical relapse of the disease in the next 6–12 months (Tibble et al. 2000).

The intention to treat analyses showed a reduction in the numerical value of FCAL levels in patients with UC but this did not reach a statistical significance (*p* = 0.076). Post-hoc analyses showed significant reductions in intestinal inflammation, which suggest that the probiotic had anti-inflammatory properties in these patients. By chance the calprotectin levels were higher at entry in the probiotic group than the placebo group, which in effect should rendered them in a higher risk group for clinical relapse, yet there were no relapses in this group and 4 in the placebo group.

Similar analyses in the CD patients showed no significant differences between probiotic and placebo treated patients and none of the patients experienced a clinical relapse.

The shortcomings of this study are that it was exploratory i.e. there was no preliminary study carried out in order to enable a robust power calculation for sample sizes and the fact that it took to a selected group of asymptomatic IBD patients that are on minimal medical treatment and such patients probably represent less that 10% of IBD patients and hence not representative of the whole group of patients with IBD.

In summary this study suggests that the probiotic Symprove may be associated with reduced intestinal inflammation in patients with UC. Further studies are however required to see if long-term ingestion of this probiotic maintains its anti-inflammatory action and whether it prevents clinical relapse of those patients that are at significant risk of clinical relapse.
